# Novel Design of Eco-Friendly Super Elastomer Materials With Optimized Hard Segments Micro-Structure: Toward Next-Generation High-Performance Tires

**DOI:** 10.3389/fchem.2018.00240

**Published:** 2018-07-20

**Authors:** Xuan Qin, Jiadong Wang, Bingyong Han, Bo Wang, Lixin Mao, Liqun Zhang

**Affiliations:** ^1^State Key Laboratory of Organic-Inorganic Composites, Beijing University of Chemical Technology, Beijing, China; ^2^State Key Lab Chemical Resource Engineering, Beijing University of Chemical Technology, Beijing, China; ^3^Engineering Research Center of Elastomer Materials on Energy Conservation and Resources, Beijing University of Chemical Technology, Beijing, China; ^4^Beijing Advanced Innovation Center for Soft Matter Science and Engineering, Beijing University of Chemical Technology, Beijing, China

**Keywords:** super elastomer, eco-friendly material, green tire, magic triangle, chain extender, cross-linking agent

## Abstract

Recently, sustainable development has become a significant concern globally, and the energy crisis is one of the top priorities. From the perspective of the industrial application of polymeric materials, rubber tires are critically important in our daily lives. However, the energy consumption of tires can reach 6% of the world's total energy consumption per annum. Meanwhile, it is calculated that around 5% of carbon dioxide comes from the emission of tire rolling due to energy consumption. To overcome these severe energy and environmental challenges, designing and developing a high-performance fuel-saving tire is of paramount significance. Herein, a next-generation, eco-friendly super elastomer material based on macromolecular assembly technology has been fabricated. Hydroxyl-terminated solution-polymerized styrene-butadiene rubber (HTSSBR) with high vinyl contents prepared by anionic polymerization is used as flexible soft segments to obtain excellent wet skid resistance. Furthermore, highly symmetrical 1,5-naphthalene diisocyanate (NDI), different proportions of chain extender, and the cross-linking agent with moderate molecular length are selected as rigid hard segments to achieve simultaneous high heat resistance. Through this approach, a homogeneous network supported by uniformly distributed hard segment nanoparticles is formed because soft segments with equal length are chemically end-linked by the hard segments. This super elastomer material exhibits excellent wear resistance and low rolling resistance. More importantly, the wear resistance, rolling resistance, and wet-skid resistance are reduced by 85.4, 42.3, and 20.8%, respectively, compared to the elastomeric material conventionally used for tire. By taking advantage of this excellent comprehensive service performance, the long-standing challenge of the “magic triangle” plaguing the rubber tire industry for almost 100 years is resolved. It is anticipated that this newly designed and fabricated elastomeric material tailored for tires will become the next generation product, which could exhibit high potential for significantly cutting the fuel consumption and reducing the emission of carbon dioxide.

## Introduction

Wheels are one of the greatest inventions in the history of human civilization and have led to significant improvements in work efficiency and liberation of productive force. The entire world mounts on wheels and advances at full speed. Tires are the only vital functional components of vehicles with direct contact with the ground, and play the crucial roles of supporting vehicle load, transferring power, braking, shock absorption, and maintaining and changing driving direction. Since rubber tires were invented, they have undergone several technical and theoretical advances; meanwhile, the rapid development of the automotive industry and highways requires tires to be one of the polymer products with maximum yield and at the highest technological level. Nevertheless, social development and environmental problems such as the increasing energy consumption, increase in safety accidents, and a vast quantity of tire waste due to the short service life give rise to a higher requirement for the development of the tire industry.

In 1980, the concept of sustainable development was explicitly proposed by “The World Conservation Strategy” (U-Nations, 1980), which has attracted extensive worldwide concern and attention in recent years. “Fossil fuel depletion” and “pollutant emissions and global warming” are the two issues that should be considered by the “sustainable energy development system” (Hammond, 2004). Owing to the rapid development of the global economy, the large use of fossil energy results in the increase of greenhouse gas emissions, sea level rise, extreme weather change, a sharp decline of biological species, and a serious threat to the environment for human survival. Therefore, the improvement in the utilization efficiency of fossil energy has become the problem that is faced and needs to be solved by the whole world. Data from the European Union indicates that transportation energy consumption accounts for more than 20% of the world's total energy consumption in the past 5 years and the fuel consumption of tires is 20–30% of that of the automobiles. Eighteen percent of the global carbon dioxide emissions are related to traffic and 24% of the carbon dioxide emissions of road vehicles are related to tires. To comply with the development trend of fuel-efficiency and safe tires with high performance, the European Union released the world-noted legislation of “Tire Labelling Regulation” in 2009 (EU, 2009). This leads to the classification of the rolling resistance and wet-skid resistance of tires. It is estimated that decreasing rolling resistance of tires by 20% leads to a 5% reduction in fuel consumption and using B-level green tires can save 20 billion liters of gasoline and reduce 50 million tons of carbon dioxide emissions every year.

At present, the rubber composites used for manufacturing pneumatic tires are prepared via vulcanization by mixing more than 10 kinds of raw materials. Therefore, the energy consumption of rubber processing is very high. Large amounts of carbon disulfide, non-methane hydrocarbons, and other organic waste are produced during the production process, and the addition of strong carcinogens (e.g., aromatic hydrocarbon oil) is required. Moreover, the constant discharge of heavy metals and dusts due to surface abrasion of rubber tires causes an adverse impact on the environment. Eighty percent of PM_10_ of the cities in the UK comes from traffic, and more than 10,000 death cases can be attributed to cardiopulmonary disease caused by PM_10_ (Karl, 2012). Furthermore, waste rubber tires cannot be recycled effectively. About 40 million tires are discarded in the UK annually (Mavroulidou et al., 2006), and the accumulation of waste tires provides a breeding place for mosquitoes and increases the risk of infectious disease outbreaks (Jang et al., 1998). Regarding the structure of tires, pneumatic tires still control the tire market. However, they suffer from the shortcomings of poor impact and puncture resistance. There is a risk of leakage or even blow-out during use, which may even lead to major accidents with loss and death. The data shows that there were 921 traffic accidents caused by tire fault in the UK in 2015, including 17 deaths and 147 serious injuries (UNECE, 2012).

The energy consumption and carbon dioxide emissions of the tire are related to the rolling resistance, whereas safety is correlated with the wet-skid resistance and solid particle pollution is affected by their wear resistance (Heinz and Grosch, 2007). These three properties are called the “magic triangle.” Traditional rubber composites are approaching a bottleneck to overcome the problem of the “magic triangle,” which restricts the development of high-performance tires.

As we reported elsewhere (Qin et al., 2018), polyurethane elastomer materials, with nano-particles chemically end-linked, have remarkably low dynamic hysteresis loss. The polyurethane features the presence of the urethane group 
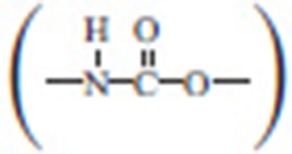
 on the macromolecular chain with a more or less frequency (Cooper and Tobolsky, 1967), which is a versatile copolymer consisting of alternating flexible soft segments and rigid hard segments (Liff et al., 2007; Jiang et al., 2015). Compared to traditional rubber composite materials, polyurethane has better wear resistance, relatively high tear strength and elongation, wide hardness range, low rolling resistance, large carrying capacity, and excellent oil and chemical resistance (Fu et al., 2016). The manufacturing process of polyurethane is simple, which leads to less consumption of environmental pollutants and the emission of waste water and gas. Besides, modified polyurethane can be made biodegradable, and hence, the recovery would be easier than that of rubber. Moreover, airless tires made from polyurethane have better safety performance and their service life is four times than that of ordinary rubber tires. The application of polyurethane in retread tires can not only reduce energy consumption but also achieve the goal of recycling development (Chiou and Schoen, 2002), which has a tremendous strategic significance of popularization. However, the undesirable wet traction, which has bearing on the glass transition temperature of the material, and poor heat resistance (Yoshida et al., 2017), which goes hand in hand with the structure of the material itself, of the preexisting polyurethane materials, bring up the inconvenience in driving security and preclude the possibility for its practical usage in the situation of high-speed and heavy-loading tires as well. Once these disadvantages are overcome, polyurethane elastomer is not only the ideal material to manufacture “green tires,” but also the best choice for realizing “green manufacturing” of tires.

To address this challenge, we put forward a new thought (Qin et al., 2018): we replace polyester polyols and polyether polyols of the soft segments of existing polyurethane materials with hydroxyl-terminated solution-polymerized styrene-butadiene rubber (HTSSBR) of designed and optimized molecule structure. HTSSBR exhibits a glass transition temperature that can be easily adjusted in a wide temperature range, and HTSSBR with relatively high levels of 1,2-butadiene and low levels of styrene possessed glass transition temperature of–25~–15°C measured by DSC. The polyurethane elastomer material with this structural designed HTSSBR acting as soft segments reveals a higher tan δ at 0°C than those of traditional materials, which means a better wet-skid resistance. Hard segments chemically link the end of equal length HTSSBR molecular chains to form a network of uniform and tunable distribution of nano-reinforcing units in the polymer matrix, which minimizes the nano-scale frictions, and the tan δ at 60°C is unusually low which means a lower rolling resistance. Meanwhile, in order to ensure the stability at very high temperature, high heat-resistant 1,5-naphthalene diisocyanate (NDI) is programmed to synthesize the innovative polyurethane elastomer material, because the low thermal conductivity of polymer may cause heat accumulation. The birth of eco-friendly next generation super elastomer material with a concurrent excellent performance of wet-skid resistance, wear resistance, rolling resistance, and dynamic mechanical properties, is indeed something to celebrate.

**Figure 1:**
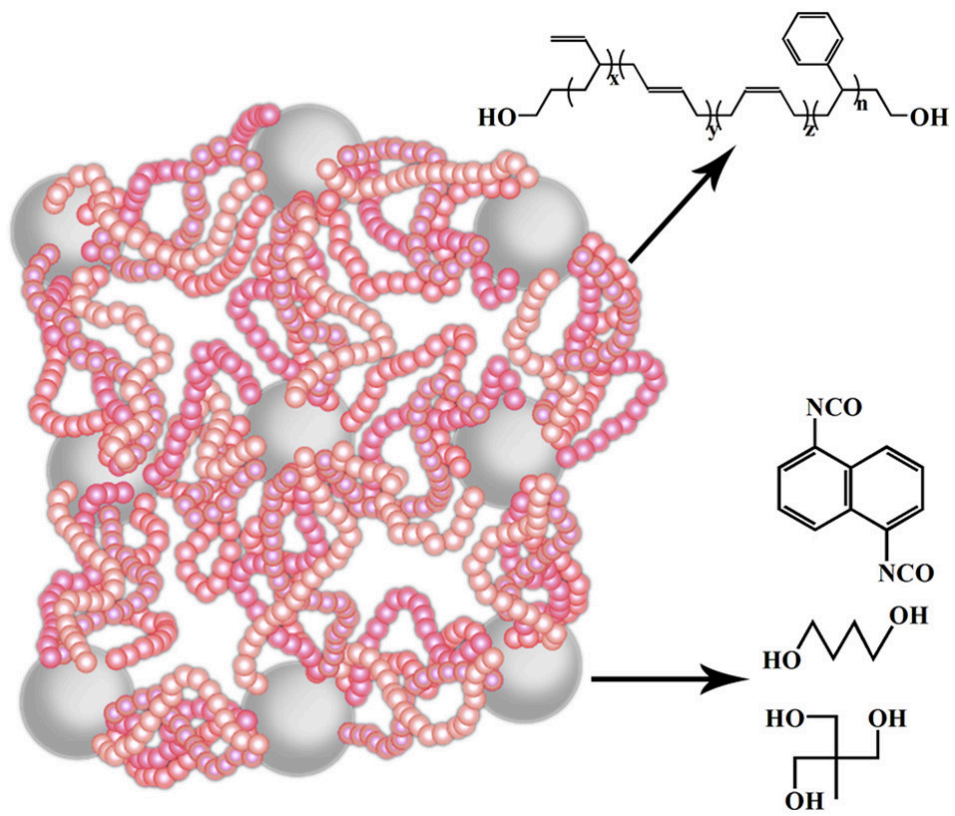
The diagram of molecular structure of our prepared elastomer material.

The properties of the super elastomer material are intensively influenced by the micro-structures. The chain extender and cross-linking agent are essential parts of the elastomer, which will determine the perfect degree of their network. Therefore, we regulated the hard segment micro-structure, principally about the ratio of chain extender and cross-linking agent of the elastomers. The effects of micro-structure change on its macroscopic properties were systematically studied by FT-IR, ^1^H-NMR, XRD, TEM, DSC, TGA, TMA, DMA, and other characterization methods. The sketch of the molecular structure of this super elastomer was shown in Figure 1.

## Experimental

### Reagents and materials

Butadiene (polymerization grade) was produced by Beijing Yanshan Petrochemical Co., China; styrene was purchased from Beijing Chemical reagents, China; THF (AR), cyclohexane (AR), and ethanol (AR) were supplied by Beijing Chemical Works, China; butyl lithium (CP) and lithium (AR) were provided by Baling Petrochemical Corp, China. NDI (AR) was supported by TCI; BDO and TMP (AR) were produced by Adamas.

### Preparation of HTSSBR

Through anionic polymerization, the hydroxyl-terminated solution-polymerized styrene butadiene rubber (HTSSBR) was synthesized using self-made dilithium anionic initiator. Considering that the anionic polymerization is highly sensitive to impurities, it is necessary to purify all the instruments and reagents. To guarantee that the polymerization was suitable, the entire reaction system was cleaned with reactive-polymer and high-purity nitrogen several times. The dilithium anionic initiator was synthesized as follows. A 1:1 (volume ratio) mixture of isoprene and cyclohexane was introduced by dripping slowly into a tetrahydrofuran solution of lithium flack protected by argon gas; the reaction was carried out at the temperature of 0°C for 6 h. After settling for 24 h, the brownish red transparent initiator solution was obtained by filtering. Next, butadiene, styrene, cyclohexane, and tetrahydrofuran were successively added into a 2 L reactor which had been thoroughly washed and purified by butyl lithium, followed by adding self-made dilithium anionic initiator. The polymerization progress lasted for 8 h at the temperature of 50°C. Then, the polymer was end-capped with ethylene oxide under stirring. After 24 h, the reaction was terminated by ethanol.

### Preparation of HTSSBR-PU

The synthesis of HTSSBR-PU was achieved by a two-step method. In the first step, the prepolymer was prepared by reacting NDI and HTSSBR at 80°C under the nitrogen atmosphere. In the second step, extender 1,4-butanediol (BDO), cross-linking agent trimethylolpropane (TMP) and the prepolymer were mixed up under high-speed stirring at 65°C for 6–8 min. At last, the sample was cast into a specific metal mold and then cured at 105°C for 24 h.

### Materials characterization

The molecular weight of HTSSBR was characterized by GPC (Waters 1525). The molecular structure was measured using 1H NMR (Bruker AV600) and FT-IR (Nicolet 8700). The micro-structure of the elastomer was measured by TEM (Hitachi JEM-3010). X-ray diffraction (XRD) with Cu-Kα radiation (λ = 0.154 nm) was performed on a Bruker D8 ADVANCE. The thermal properties were tested by TGA (Mettler-Toeledo), DSC (Mettler-Toeledo STARe), TMA (TA Q800), and DMA (TA Q800). The mechanical performances of the elastomer were conducted by the universal testing machine (LRX Plus/LLOYD LRX/MTS CMT4104), Akron abrasion tester (MINGZHU MZ-4061) and a friction coefficient tester (LEIYUN BM-III). The detailed information of material characterizations is offered in the Supplementary Materials.

## Results and discussion

HTSSBR was successfully synthesized via anionic polymerization. By analyzing ^1^H NMR and FT-IR spectroscopy, the content of 1,2-butadiene, 1,4-butadiene and styrene were 61.56%, 14.69 and 23.75%, respectively. The glass transition temperature is −22.47°C as expected. The molecular weight is ~3,000 and with a narrow distribution.

**Figure 2:**
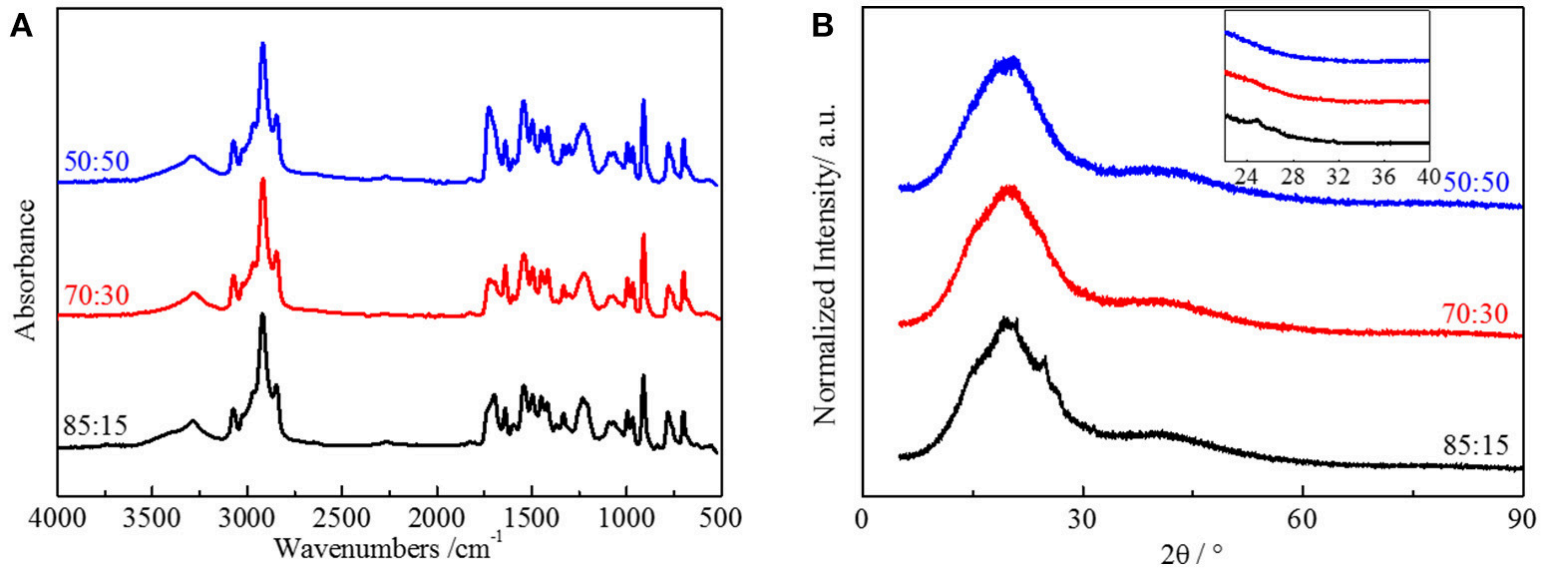
**(A)** FT-IR spectra and **(B)** XRD spectra of HTSSBR-PU with different ratios of chain extender to cross-linking agent.

To demonstrate our thought, we emphasized polyurethane materials using HTSSBR as soft segments with different ratio of cross-linking agent and chain extender. The chemical content of the elastomer is a critical factor for the performance of the material. FT-IR spectroscopy (Figure 2A) was used to support that the synthesis progress was successful. The sample is designated as A:B, where A and B denote the mass percentages of chain extender and cross-linking agent, respectively. Taking the sample 50:50 as an example, the broad absorption at 3,290 cm^1^ corresponds to the N-H bonding. The absorptions from 1,740 to 1,700 cm^−1^ correspond to the stretching vibration of C=O. The absorption at 2,280–2,255 cm^−1^, the witness of –NCO, indicates the complete reaction of NDI. There is redshift of the absorption peaks of N-H and C=O, attributed to the hydrogen bonded amino group and the carbonyl group. By using peak-fitting software and imitating procedure by the Levenberg-Marquardt algorithm, hydrogen bonding degree calculated by peak areas is summarized. Degrees of ordered and disordered hydrogen bonding, and the total degree of hydrogen bonding decrease with the increasing content of the cross-linking agent (And and Painter, 2007). In this system of polyurethane, the soft segment made up of styrene and butadiene does not include strong electronegativity elements, making the hydrogen bonding only form between the hard segments (Harthcock, 1989; Król, 2007). The decrease of microtacticity of hard segments, which is lowered by the trifunctional trimethylolpropane, is the main reason for the reduction of hydrogen bonding. Compared with the difunctional 1,4-butanediol, the structure formed by TMP is more rigid and irregular, making the hydrogen bonding harder to form. The degree of ordered and disordered hydrogen bonding both decreases from 52.39 to 45.41% and from 22.64 to 18.30% respectively, resulting in the total degree of hydrogen bonding decreasing from 74.03 to 63.71%. The crystallization property of hard segments in the material, which is also associated with the molecular structure, is also influenced by the reduction of hydrogen bonding degree. X-ray diffraction (XRD) was performed to explore the crystallization of the elastomer (Figure 2B). There are dispersing diffraction peaks and a sharp diffraction peak (2θ≈20°). The dispersing diffraction peak is formed due to the long-range disorder of the elastomer, and the sharp diffraction peak is formed by the short-range ordered crystal area. The shape of the dispersing diffraction peaks is almost identical, whereas the strength of sharp hard segment diffraction peak decreases and the degree of crystallization decreases from 7.27 to 1.22% with the ratio of chain extender to cross-linking agent reduces. As the main content of hard segment, NDI is a kind of isocyanate with good symmetry, which benefits from the regular structure of molecular chains and allows the hard phase to have crystallization. Crystallization formed by hard segment units is short range order, and the whole elastomer remains long range disorder. As a tri-functionality cross-link agent, TMP is not as symmetrical as BDO. This influences the formation of short-range order and partly long-range order structure, so the degree of crystallization will decrease with the accretion of TMP (Seefried and Critchfield, 2010).

**Figure 3:**
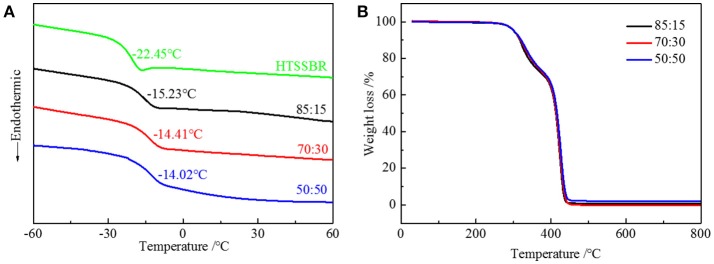
**(A)** DSC curves and **(B)** TGA curves of HTSSBR-PU with different ratios of chain extender to cross-linking agent.

**Figure 4:**
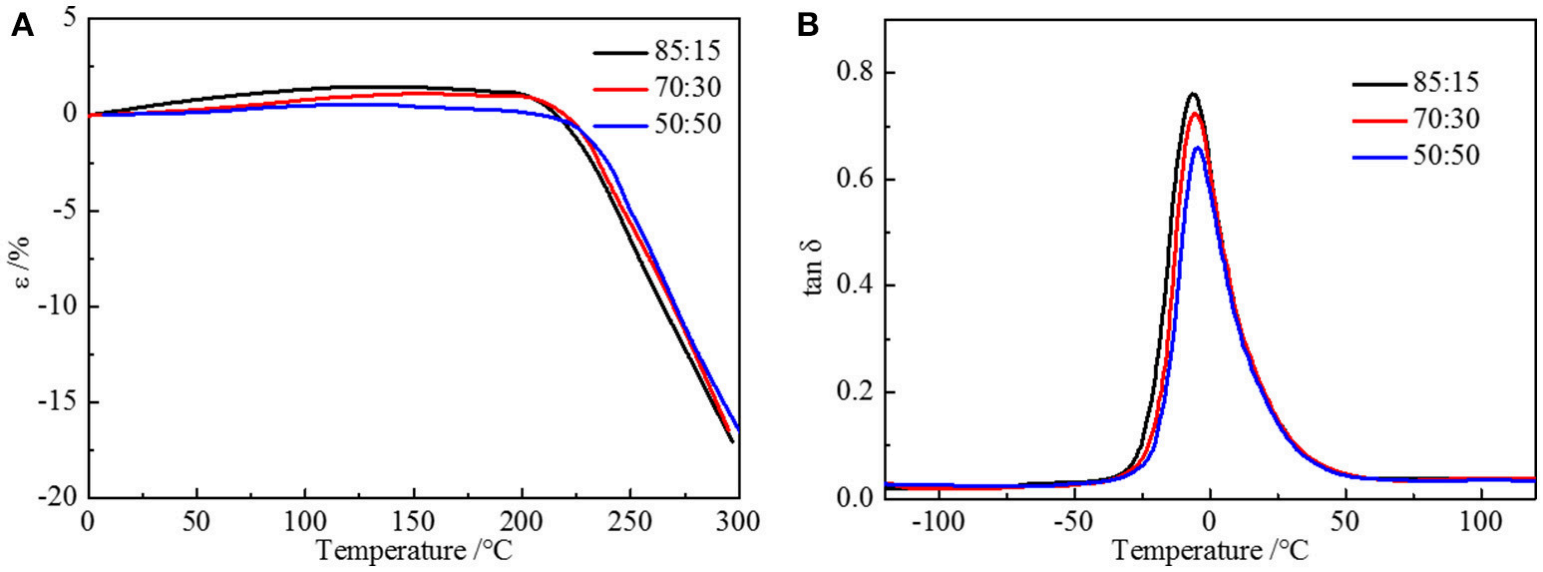
**(A)** TMA curves and **(B)** DMA curves of HTSSBR-PU with different ratios of chain extender to cross-linking agent.

Due to the difference in polarity between the soft and the hard segments, the micro-phase separation and chemically end-linked nanoparticles are generated uniformly through the self-assembly progress. The thermal and mechanical properties are mainly supported by the hard segment nanoparticles; therefore, it is of vital importance to study the effect of the structure of hard segment. The thermal properties were acquired through thermogravimetric analysis and differential scanning calorimetry. Glass transition temperature (Figure 3A) of HTSSBR is−22.45°C, and glass transition temperature increases as the ratio of chain extender to cross-linking agent reduces. As described in the theoretical formula of glass glass transition temperature:

$$\begin{array}{l} T_{g_{x}}=T_{g}+K_{x}\rho \end{array}$$

where *T*_*gx*_ is the glass transition temperature of the cross-linked polymer, *T*_*g*_ is the glass transition temperature of the origin polymer, *K*_*x*_ is the characteristic constant, and ρ is the cross-linking density. As the cross-linking density increases, the glass transition temperature of the cross-linked polymer will rise correspondingly. Adding more crossing-linking agent into the system increases the chemical cross-linking density, and the mobility of molecular chains is restricted by the three-dimensional network restricts, which will raise the glass transition temperature. The thermal decomposition progress (Figure 3B) of PU is composed of two-stages, including hard segment and soft segment dissociation (Petrović et al., 2010), successively. The amount of final residual carbon is almost equal. The weight loss rate of two stages is the same as the weight ratio of the hard and soft segment, which indicates the complete reaction as well. The initial thermal decomposition temperature is about 300°C. The changing ratios of chain extender to cross-linking agent have little effect on the two-stage weight loss. The decomposition is mainly caused by chemical bond rupture, while the network structures formed by TMP and BDO have a slight influence on chemical bonds, so the thermal destruction temperature increases slightly. Thermomechanical analysis (TMA) was applied to further investigate the thermal properties of the elastomer. The softening temperature is defined as the critical temperature when the elastomer having a reduction in thickness which leads to a sudden decline in modulus (Lee and Ko, 1993). The softening temperature and expansivity were measured (Figure 4A). The disentanglement of hard segment is relieved by cross-linking structure at high temperature, which raises the softening temperature. At the same time, with the content of cross-link agent increasing, the deformation is more restricted by the network structure with temperature increases. By comparing with our previous work, the increase of the hard segment content has more effect on softening temperature than that of the ratio of the cross-linking agents to the chain extender. The explanation is as follows: these two factors both contribute to the cross-linking points of the structure. The difference is that hard segment, as physical cross-linking points, mainly forms the well-defined micro-phase separation due to hydrogen bonding and polarity of hard and soft segment, however cross-linking agents have mainly impacted the hard segment phase and have no direct correlation with micro-phase separation. Additionally, the hardness of the elastomer is slightly influenced by the changing the ratio of the chain extender to the cross-linking agent, which varies from 70 to 73. In a micro structure, the hardness of the partial area is increased by the network structure formed by tri-functional cross-linking agent. As analyzed earlier, the macro properties are mildly affected by the cross-link in hard segment phase, so there is a slight increase in hardness. Dynamic mechanical analyzer (Figure 4B) was used to test the mechanical properties of the elastomer (Dong and Cooper, 1971). The area of loss peaks is reduced as the ratio of chain extender to cross-linking agent decreases. The tan δ 0°C, which is also known as the wet-skid resistance, is influenced by change of cross-linking density. The increase in cross-linking density limits the mobility of molecular chain between cross-link points, which will reduce the loss of energy, resulting in the decrease of peak area. The temperature of the loss peak moves to high temperature, as evidenced in DSC.

**Figure 5:**
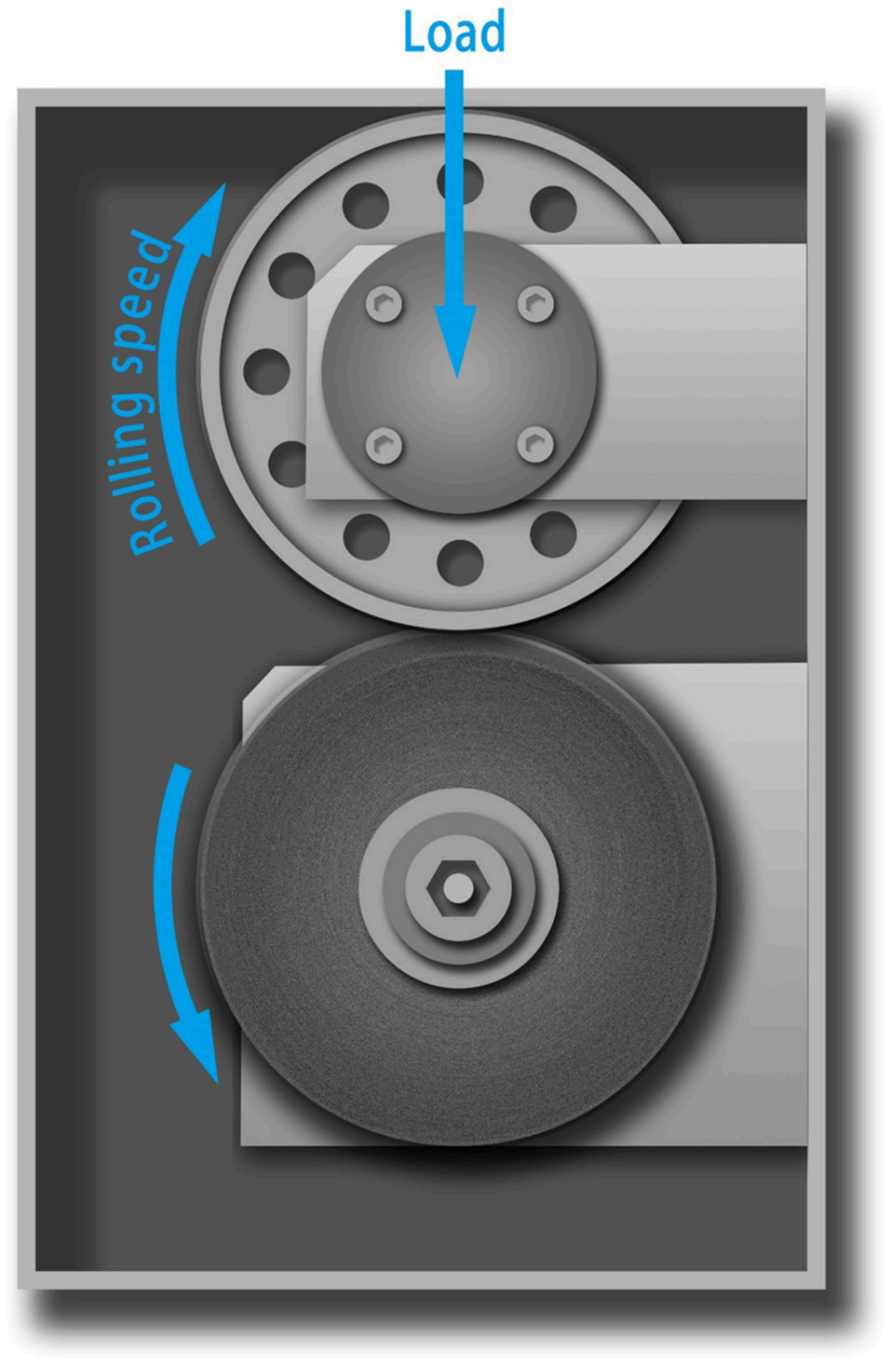
The schematic diagram of the rolling resistance test machine (RSS II model).

**Figure 6:**
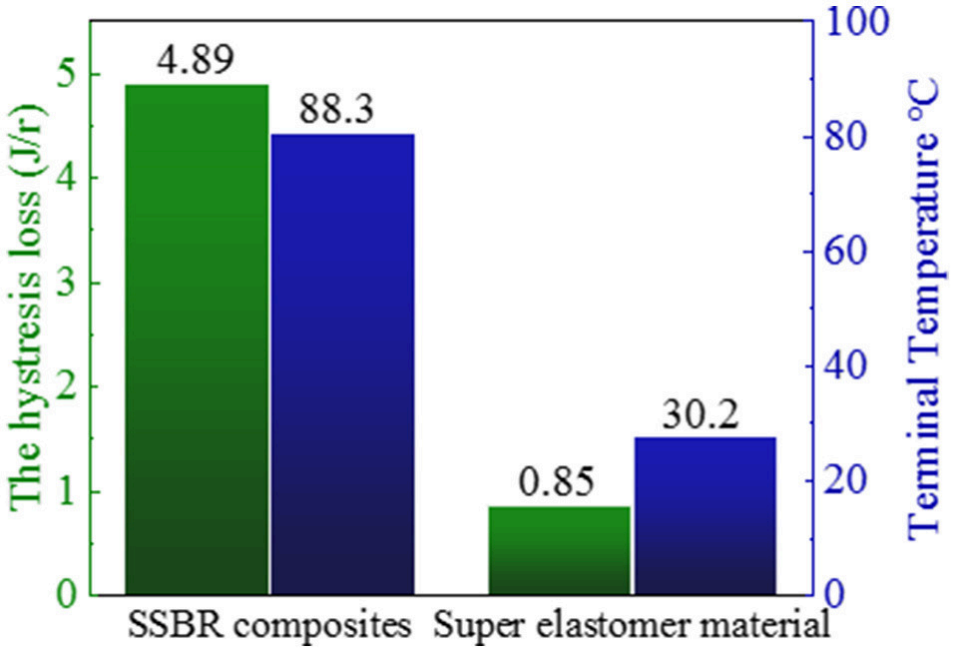
The hysteresis loss and terminal temperature of SSBR composites and our prepared super elastomer material.

**Figure 7:**
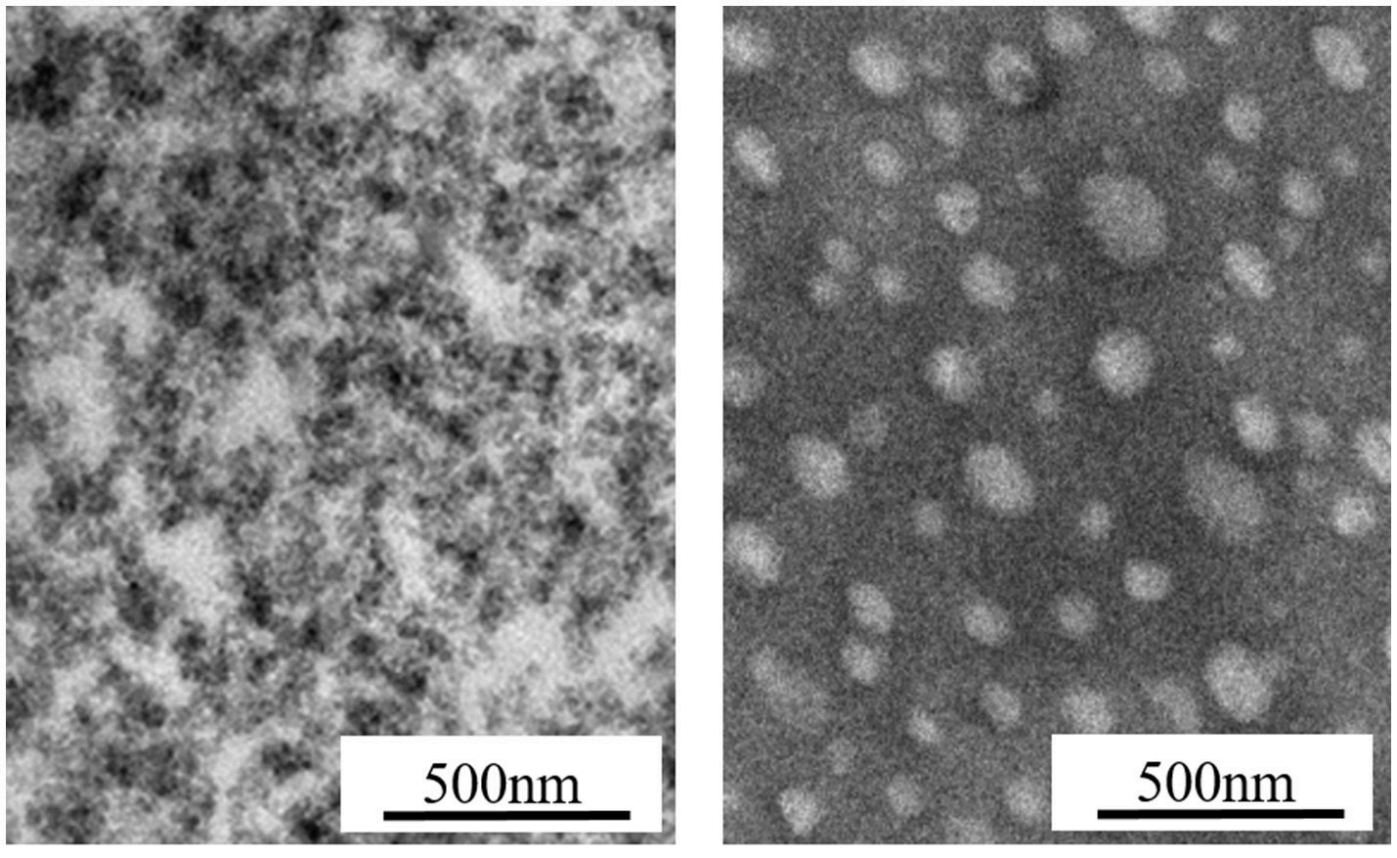
TEM images of SSBR composites and HTSSBR-PU.

**Figure 8:**
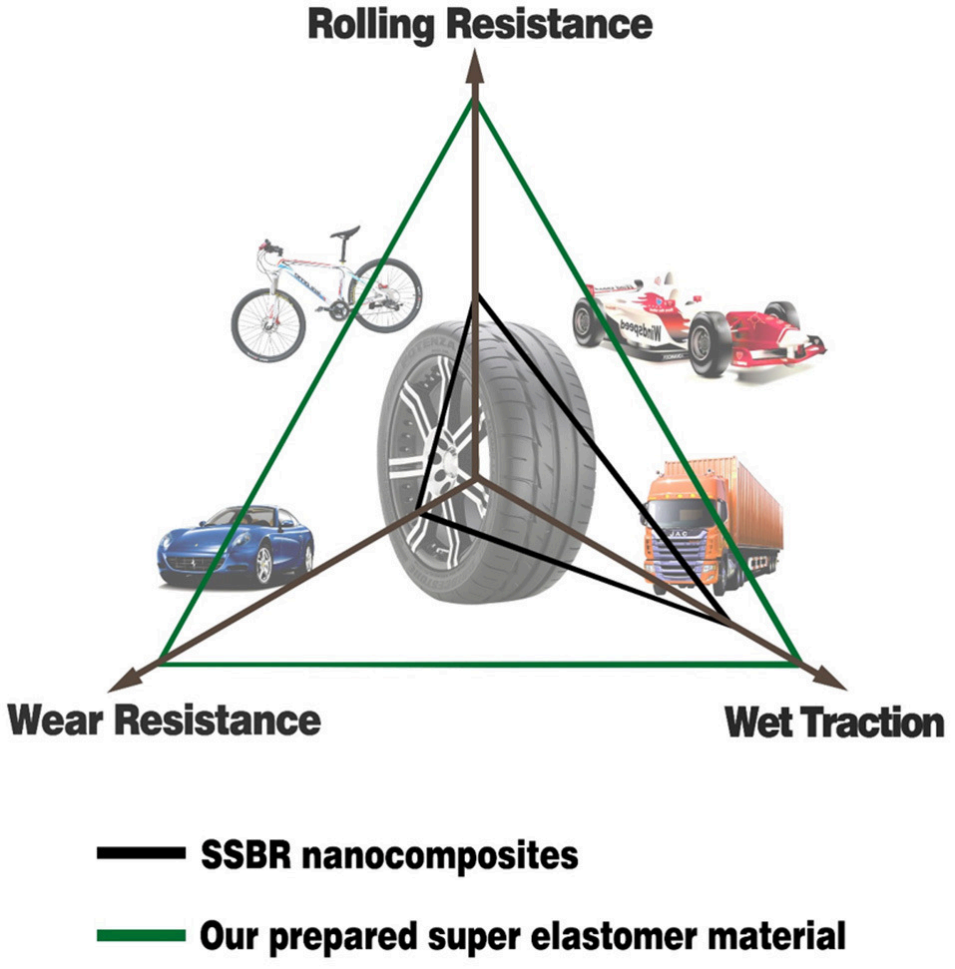
The “Magic triangle” performance comparisons between SSBR composites and our prepared super elastomer material.

The above performances of HTSSBR with different ratios of chain extender and cross-linking agents completely match the requirement of a tire material (Prisacariu, 2011). Comparison between the sample with relatively better performances and SSBR composites are made to highlight the excellent mechanical properties. The general mechanical properties comparison are given in Table 1. The tensile strength is 22.3 MPa, compared with SSBR composites (16.1 MPa), an increase of 38.5%. The elongation at break is 481%, while the elongation of SSBR composites is 365%. Moreover, the wear resistance of HTSSBR is much better, with Akron abrasion loss at 10.8 mm^−3^, while the Akron abrasion loss of SSBR composites is 74 mm^−3^, which is much larger than Akron abrasion loss of HTSSBR. Tires with better wear resistance means less weight loss at work and longer service life, which can not only save the cost but also protect the environment. The exceptional wear resistance of the elastomer makes it an environmental-friendly material. In comparison with HTSSBR and SSBR composites, our PU elastomer shows apparent improvement in mechanical properties. However, before this elastomer comes to practical application, there are still several properties to be discussed. Hysteresis loss, which is also called dynamic energy loss, is one of the most important properties of dynamic elastomers products. As usual, the hysteresis loss dissipates in the form of heat, so it is known as rolling resistance. The dynamic energy loss of tires (Futamura, 1991), turning into heat accumulation, will raise the working temperature of the tires, which could affect the mechanical properties of the tire and have a potential safety hazard. At the same time, rolling resistance determines the fuel assumption of tires. Therefore, it is of great importance to develop tires with low dynamic energy loss. In order to reflect the property of the HTSSBR-PU, a comparison between HTSSBR-PU and conventionally SBBR composites used for tire used was invested to discuss the mechanical performance of the HTSSBR-PU. Based on the theory of the viscoelasticity of elastomer, a machine (RSS-II model, Figure 5) was designed to measure the rolling resistance. The dynamic energy loss can be measured by the viscoelasticity response of the elastomer in the rotating progress of the material sampler on the roller. The dynamic strain and stress (Willett, 1973) given as ε(*t*) = ε_*a*_ sin (ω*t* − δ) and σ(*t*) = σ_*a*_ sin ω*t*, respectively. The energy loss under a specific controlled energy cycle is give as follows

$$\begin{array}{l} H=\int_{0}^{2\pi /\omega }\sigma \left(t\right)\frac{d\varepsilon \left(t\right)}{dt}dt=\pi \varepsilon_{a}\sigma_{a}sin\delta \approx \pi \varepsilon_{a}\sigma_{a}tan\delta \end{array}$$

where ω is the frequency, ε_*a*_ is the amplitude of strain, σ_*a*_ is the amplitude of stress, δ is the phase difference between stress and strain, and *tanδ* is the loss factor. After the test cycling (Figure 6), the hysteresis loss of HTSSBR-PU is measured to be 0.85 J/r, which is significantly lower than the hysteresis loss of the SSBR composites (4.89 J/r). Due to the heat accumulation caused by the dynamic energy loss, the temperature rise of HTSSBR-PU is 30.2°C, but the temperature rise of SSBR composites incredibly reached 88.3°C. To simulate the reality viscoelastic response relation with alternating stress of the materials under the working conditions, DMA with the strain ε at 7% and the frequency at 10 Hz was performed. The 60°C tan δ of the SSBR composites (0.130) is larger than the 60°C tan δ of HTSSBR-PU, which could further prove that the latter have lower rolling resistance. In addition, the pendulum-type friction coefficient reflects that the HTSSBR-PU lost less energy during the friction process, illustrating the low rolling resistance of HTSSBR-PU. As Figure 7 shows, the microstructure of SSBR composites is uneven due to the severe conglomerating of silica nanoparticles, while homogeneous phase separation in HTSSBR-PU is generated by the self-assembly progress of soft and hard segments. At the molecular level, the viscoelasticity of the SSBR composites is mainly based on the polymer blend of SSBR/BR, the size, and distribution of nano-particles. The vulcanization and the nano-particles provide the essential chemical cross-linking points, but there will be the problem of combination between the particles and the polymer and uneven distribution of polymer and filler. The system of HTSSBR-PU is totally different in these aspects; the synergy of the network is supported by the soft segment with uniformly dispersed molecular weight and the even distribution of physical cross-link points provided by the micro-phase separation (Chen and Hsu, 1990; Cho et al., 2013), which leads to noticeable diminution of hysteresis loss and decrease of heat accumulation. The well-performed rolling resistance can also obviously reduce fuel consumption, which effectively reduces the carbon emission. Additionally, the molding process of HTSSBR-PU, which does without mixing and vulcanization, is much more straightforward and more efficient than the preparation technology of SSBR composites. To highlight our super elastomer material more intuitively, “Magic triangle” performance comparisons with SSBR composites are expressed in Figure 8. The markedly enlarged “Magic triangle” area elucidated that our super elastomer has the highest comprehensive performance. It is now well-established that our super elastomer is a kind of advanced material with fantastic performance and inestimable application prospects.

**Table 1:** The comparison between SSBR composites and our prepared super elastomer material on mechanical properties.

	**SSBR composites^a)^**	**HTSSBR-PU**
Tensile strength /MPa	16.1	22.3 (+38.5%)
Elongation at break/%	365	481 (+31.8%)
Tensile stress at 300%/MPa	12.9	15.1 (+17.1%)
Tear strength /kN·m^−1^	40.6	45.8 (+12.8%)
Compression heat build-up/°C	20.9	16.2 (−22.5%)
Akron abrasion loss /mm^3^	74.0	10.8 (−85.4%)
60°C tan δ(strain 7%, frequency 10 Hz)	0.130	0.075 (−42.3%)
Pendulum-type friction coefficient	24	29 (+20.8%)

a) *Formulas of contrast sample SSBR composites are shown as follows: SSBR 96.3, BR 30.0, Silica 70.0, Bis [3-(triethoxysilyl)propyl] tetrasulfide (TESPT) 6.3, Stearic acid (SA) 2.0, ZnO 3.5, N-tert-Butyl-2-benzothiazolesulfenamide (TBBS) 2.0, diphenyl guanidine(DPG) 2.0, N-1,3-dimethylbutyl-N′-phenyl-p-phenylenediamine (Antioxidant 4020) 1.0, S 1.7*.

## Conclusions

In conclusion, we designed and synthesized a super elastomer material by combing the HTSSBR and NDI, and the properties influenced by the ratio of chain extender and cross-link agent are discussed in particular detail. The excellent mechanical properties of the elastomer are provided by the micro-phase separation structure and hard segment nanoparticles, and the structure of hard segments are influenced by the chain extender and cross-link agent. As the content of cross-link agent increases, the short-range order structures are affected and the degree of crystallization decreases. The tri-functional TMP raises the cross-link density of the hard segment, which increases the softening temperature and reduces the expansivity. The network structure in the hard segment has a light impact on the micro-phase separation and heat resistance of the material. Compared with commercial SSBR composites, the super elastomer material has advantages in most aspects, especially the excellent rolling resistance and wear resistance, which help to decrease the carbon dioxide emission. Our prepared elastomer material perfectly matches the requirement of “green tire,” and it provides a new idea for further development excellent energy-saving and eco-friendly advanced elastomers.

## Author contributions

XQ, JW, and BW contribute to the experiment process, samples characterization, data analysis, and paper preparation. BH, LM, and LZ are mainly responsible for the design of experiment, data analysis and paper revision.

### Conflict of interest statement

The authors declare that the research was conducted in the absence of any commercial or financial relationships that could be construed as a potential conflict of interest.
