# Supplementary material for: Novel Design of Eco-Friendly Super Elastomer Materials With Optimized Hard Segments Micro-Structure: Toward Next-Generation High-Performance Tires
Source: Front Chem. 2018 Jul 20;6:240. doi: 10.3389/fchem.2018.00240 (PMC6062621; doi:10.3389/fchem.2018.00240)
Supplement: Supplementary file 1 [file Presentation_1.PDF]

The details of material characterization: ^{1}H-NMR spectra were characterized by a Bruker spectrometer (AVANCE-Ⅲ 400 MHz) at 25℃ using deuterated chloroform ($CDCl_3$) as the solvent. FT-IR spectra were recorded on the Bruker TENSOR II at a signal-to-noise ratio of 32,000:1 and a resolution of 0.5 cm^{-1}. XRD experiments were conducted on a Bruker D8 ADVANCE with a rotating anode emitting X-rays Cu-Kα radiation (λ=0.154 nm) and were used to measure the crystallization of the elastomers. 2D XRD patterns were measured by using an image plate after an exposure time of 1 h. After the material was made to be a nanosheet with a freshly cut surface at -60 ℃ using an ultramicrotome (Leica EMUC7) and dyed by OsO4 to distinguish the soft segement to hard segment, TEM analysis was performed on a JEOL JEM3010 at the acceleration voltage of 200 kV. Differential scanning calorimetry (DSC) were performed using a METTLER-TOLEDO STAR^e system. Samples were heated from room temperature to 150 ℃ and cooled to −100°C to eliminate the heat history, and ramped up to 150 ℃ at the rate of 10 ℃ min^{-1}. Thermogravimetric analysis (TGA) was carried out using a METTLER-TOLEDO STAR^e system under a nitrogen atmosphere. The heating procedure for the samples was from 25 ℃ to 800 ℃ at a rate of 10 ℃ per min. Dynamic mechanical analyze (DMA) was performed on a 01 dB-metravib VA3000 with a temperature sweep from -125 ℃ to 125 ℃ to measure the loss factor tan δ, the strain ε was 0.1% and the frequency F was 1 Hz. A Bruker Q400 was used to test the thermomechanical properties from 0 ℃ to 250 ℃ at the heating rate of 5 ℃ min^{-1}. Tear tests and Tensile were performed according to ISO standard 34-1:2004 and ISO standard 37:2005, respectively, by using a universal testing machine (LRX Plus/ LLOYD LRX/ MTS CMT4104). The abrasion test, according to

ISO standard 5470, was conducted on a MINGZHU MZ-4061 Akron abrasion tester. The rolling resistance test to measure the power loss and temperature increase of the wheels were carried out on a WANHUI RSS-II tester with a 30 kg loading at the rotate speed of 600 rpm. After the sample was processed by sodium bicarbonate and isopropanol for 24 h, a pendulum friction coefficient tester (LEIYUN BM-III) was used to measure the pendulum-type friction coefficient.
